# Development of Novel Formulation for Sustained Release of Drug to Prevent Swainsonine-Containing Plants Poisoning in Livestock

**DOI:** 10.3390/ani13162646

**Published:** 2023-08-16

**Authors:** Bo Li, Congsheng Zhang, Yiru Zhu, Pinzhi Sun, Shangrui Fan, Weina Wang, Yanan Tian, Hao Lu

**Affiliations:** 1 College of Veterinary Medicine, Northwest A&F University, Yangling 712100, China; libo199800@163.com (B.L.); 17797250885@163.com (C.Z.); stephzhu86@gmail.com (Y.Z.); sunpinzhi@nwafu.edu.cn (P.S.); 18092625381@126.com (S.F.); wangweina38@163.com (W.W.); 2 Department of Veterinary Physiology and Pharmacology, College of Veterinary Medicine, Texas A&M University, College Station, TX 77843, USA; ytian@cvm.tamu.edu

**Keywords:** “Jifang E”, sustained-release injection, swainsonine-containing plants, security evaluation

## Abstract

**Simple Summary:**

Swainsonine-containing plants are a poisonous plant widely distributed in grasslands around the world, poisoning by it can cause livestock deaths and economic losses to herdsmen. We have developed a sustained-release injection for the prevention of poisoning by swainsonine-containing plants. Compared to other formulation, e.g., powders and pills, injections have the advantage of convenient administration with easy dosage control. The injection uses poloxamer as the sustained-release matrix and can be solidified into a gel at physiological temperature. We have shown that this formulation is safe and can maintain an effective blood drug concentration for up to 5 days, providing a new approach for preventing swainsonine-containing plants poisoning livestock.

**Abstract:**

Swainsonine-containing plants contain swainsonine which has been shown to cause neurological signs and pathological changes in farm animals. It causes a large number of livestock poisonings every year resulting in economic losses to the livestock industry. At present, “Jifang E” is used in the prevention of swainsonine-containing plants poisoning livestock, and the preventive effects have been well-documented. However, “Jifang E” is typically administered in drinking water, making it difficult to control the administered dosage, because of feeding difficulties and it may cause certain side effects that are unique to the water-dissolved powder. To overcome these difficulties, we developed a temperature-sensitive gel for injection and the optimal ratio of each formula of sustained-release injection is P407 (24%), P188 (6%), Vitamin C (1%), PEG4000 (0.5%), and “Jifang E” (10%). Our results suggest that novel formulation makes the micellar system more stable and the particles are uniformly dispersed. Colloidal morphological studies showed that each group formed a homogeneous pore structure after gelling, and the structure became more dense with the addition of “Jifang E”. The rheological study shows that “Jifang E” is a pseudoplastic fluid, and the addition of “Jifang E” reduces the viscosity of the formula, which is beneficial to the injection. In vitro and in vivo release rate studies have shown that the effective concentration of “Jifang E” can be maintained for 3 to 5 days. The acute toxicity test in SPF Kunming mice showed that its LD_50_ was 828.323 mg/kg, with confidence limits of 676.706–1013.911 mg/kg, which is a safe dosage (LD_50_ > 200 mg/kg). There were no observed reactions of muscle irritation or subcutaneous tissue irritation with the dosage used for New Zealand rabbits. In summary, we successfully developed the sustained-release injection formulation of “Jifang E” for the prevention of swainsonine-containing plants poisoning livestock, which provides the basis for subsequent field extension trials and the further study of its detoxification mechanism.

## 1. Introduction

Swainsonine-containing plants are widely distributed throughout the world including western United States and in pastures in Asia and South America. The main toxic component of swainsonine-containing plants is swainsonine (SW), a secondary metabolite of plant endophytic fungi, which is water-soluble and has a semi-chair structure similar to the structure of α-mannosidase substrate, and therefore has a competitive inhibitory effect on α-mannosidase [1], leading to the accumulation of oligosaccharides and glycoproteins in the affected cells causing vacuolar degeneration. Swainsonine-containing plants are mainly neurotoxic and have also been shown to have reproductive and embryonic developmental toxicity, causing neurological dysfunction, reduced reproductive capacity, and reduced production performance in livestock [2,3]. The short half-life of swainsonine (elimination half-life of 20 h) hinders the validity of serum swainsonine concentration as a biomarker, and histopathology is usually the best diagnostic method for swainsonine-containing plant poisoning [4].

“Jifang E” is a drug jointly developed by Northwest A&F University and Qinghai University to prevent swainsonine-containing plants poisoning livestock, and this drug can significantly delay the time taken for sheep to be poisoned by swainsonine-containing plants [5]. Metal ions make up the majority of “Jifang E”, as swainsonine competitive sexual inhibition α-mannosidase activity is the poisonous mechanism of swainsonine-containing plants, which these metal ions can enhance via α-mannosidase activity [6]. The common “Jifang E” application is via daily feeding through drinking water (1 mL/kg·bw), which has the disadvantages of large inter-individual variation and difficulty in controlling the amount administered and has certain side effects through rapid absorption. The alternative pill formulation is time-consuming, laborious, and subject to regurgitation, thus affecting the preventive effect of the drug. Therefore, there is a need to develop a new type of formulation, which can be easily administered and can achieve the effect of sustained release. Sustained-release formulation technology applied to animal drugs can reduce the number of doses in animals, maintain a stable effective drug concentration over a longer period of time, avoid peaks and valleys that occur with multiple doses, reduce toxic side effects, reduce the number of doses, and save costs [7]. The temperature-sensitive gel systems are widely used because these systems do not require any external reagents or organic solvents to form the gel. Hydrogen bonding and polymer interactions occur between molecules, and gels are formed with changes in temperature [8,9]. Poloxamer is a nonionic surfactant and also a commonly used excipient in the prescription of temperature-sensitive in situ gels [10]. Among them, poloxamer 407 (P407) and poloxamer 188 (P188) are the most commonly used excipients in the drug sustained-release matrix in this type of matrix. Poloxamer is a triblock copolymer of polyethylene oxide (PEO) and polypropylene oxide (PPO) with amphiphilic properties, depending on their PEO/PPO weight ratio. Poloxamer 407 (P407) is more commonly used with a relative molecular mass of 12.6 kDa, of which PEO accounts for 70% and PPO for 30%. The polymer molecule is dehydrated at the critical micelle temperature when the hydrophobic poly (propylene oxide) (PPO) block on its chain breaks the hydrogen bond, forming a spherical micelle with a lipophilic PPO chain as the inner core and a hydrophilic poly (ethylene oxide) (PEO) chain as the outer shell, and as the temperature increases, the entanglement and stacking between the micelles intensifies and gelling occurs [11]. The good thermal reversibility of the aqueous solution containing P407 is due to the fact that the P407 molecular structure is surrounded by a hydrated layer at low temperature, and the increase in temperature causes hydrogen bonding between the solvent and the hydrophilic chains of the copolymer to break, promoting the formation of micelles. At room temperature, the gel system is a liquid, and after injection, a solid gel is formed at body temperature. Formulations containing P407 at 15% to 30% concentration form gels at body temperature [12,13]. Since its approval by the FDA as an inactive ingredient in intravenous, inhalation, oral liquids and suspensions, and ophthalmic or topical formulations, it has been used for its physiological tolerability, low toxicity, biocompatibility, ease of preparation, and compatibility with other drugs. It has been widely used as a drug delivery system due to its good physiological tolerance, low toxicity, biocompatibility, ease of preparation, and good compatibility with other drugs and pharmaceutical excipients [14,15].

The objectives of this study were to develop a temperature-sensitive in situ gel formulation of “Jifang E”, to optimize the ratio of each component according to the gelling temperature and the nature of the drug, to evaluate its physicochemical properties, and to determine its in vitro release rate. In order to evaluate the safety and in vivo slow-release effect of “Jifang E” sustained-release in situ gel, the acute toxicity and muscle stimulation tests and pharmacokinetic tests of “Jifang E” in situ gel were performed. Our results showed that the temperature-sensitive gel based on poloxamer can achieve injectability and a sustained release of “Jifang E” in vivo, and it has good biocompatibility and is safe, consequently, it shows the potential to be a new choice for the prevention and treatment of locoweed poisoning in livestock in future.

## 2. Materials and Methods

### 2.1. Materials and Animals

We acquired “Jifang E” (prepared in the laboratory of Northwest A&F University), Poloxamer 407, Poloxamer 188, vitamin C (Yuanye Biological Company, purchased from Shanghai, China), PEG 4000 (Solarbio, purchased from Beijing, China), dialysis bags (MD 8000–12,000) (Solarbio, purchased from Beijing, China), PBS (pH 7.36, homemade in the laboratory), and a determination kit for “Jifang E” drug content in plasma (Nanjing Jiancheng Institute of Biological Engineering, purchased from Nanjing, China).

Healthy SPF Kunming mice and New Zealand rabbits were purchased from Chengdu Dashuo Experimental Animal Co., Ltd. (Chengdu, China). All experimental procedures in this study met the requirements of the animal ethics committee of Northwest A&F University and complied with the animal welfare provisions formulated by the Institutional Animal Care and Use Committee (18 July 2021).

### 2.2. Formulation Screening of “Jifang E” Sustained Release In Situ Injection

All formulations were configured using the cold dissolution method, and the optimum amount of each ingredient was determined by screening the gelling temperature of the in situ gel of “Jifang E”. Briefly, P407, P188, “Jifang E”, and polymer additives were completely dissolved in deionized water and stirred continuously with a magnetic stirrer until a homogeneous solution was obtained, which was placed at 4 °C for more than 24 h.

### 2.3. Gelation Temperature Test

The gelling temperature was determined by the test tube inversion method: 0.5 mL of the blank gel was added to a 1.5 mL transparent centrifuge tube, placed in a water bath at 15 °C, and the temperature was continuously increased at a rate of 0.5 °C/min until gelling occurred and the temperature at this time was the gelling temperature. Each sample was measured three times and the average value was taken.

### 2.4. Detection of Micelle Hydrodynamic Diameter and Its Distribution

In order to simulate the behavior of micelles at room and physiological temperatures, the hydrodynamic diameter and the mean size distribution of micelles were determined with a nano-laser particle sizer (Malvern Instruments, Malvern, UK) at a fixed angle of 173° and temperatures of 25 °C and 37 °C, respectively. Preparations were performed according to the requirements of the instrumental tests, and colloidal solutions of different compositions were analyzed with or without additives as well as drugs, and for each sample, all measurements were taken at least three times.

### 2.5. Rheological Analysis

Rheological analysis of hydrogel samples was performed using a rotational rheometer (Waters, Milford, MA, USA). The change in viscosity (η), the change in elastic modulus (G′), and viscous modulus (G″) of the gel samples were measured at different formulations in the temperature range of 15 to 45 °C. The change in viscosity (η) of the “Jifang E” sustained-release in situ gel was measured at 25 °C and 37 °C with increasing shear rate (0.1 to 1001/s), and the change in viscosity (η) of the “Jifang E” sustained-release in situ gel was measured at 37 °C with increasing oscillation frequency (0.1 to 1001/s). The change in viscosity (η) was measured at 37 °C, and the change in elastic modulus (G′) and viscous modulus (G″) of the “Jifang E” in situ gel was measured at 37 °C with increasing oscillation frequency (0.1 to 100 Hz). All measurements were performed using an aluminum Perle plate (40 mm diameter).

### 2.6. Morphological Analysis

Characterization of P407, P407 + P188, P407 + P188 + additive, P407 + P188 + additive + ”Jifang E” after gelling in SEM was observed using a NanoSEM-450 field emission scanning electron microscope. Since the samples contained more liquid components, the conventional vacuum drying method could not achieve accurate results, so the Cryo-SEM was used. The specific operation steps were: sample taking; sample loading; sample holder loading to the transfer device; sample pre-cooling (insertion of liquid nitrogen slurry); transfer under vacuum; airlock docking with the preparation chamber; fracture, sublimation etching, and gold spraying; and transfer to the electron microscope chamber (SEM observation and photography), the whole Cryo-SEM operation is performed under the premise of “cryogenic freezing” and “vacuum state”.

### 2.7. In Vitro Dissolution and Drug Release Assays

Membrane-free release with membrane-free dissolution is the precise aspiration of an appropriate amount of in situ gel preparation in a pre-weighed mass of 10.0 mL cillin vial, which is then weighed so that approximately 5 mL of in situ gel is added. This cillin bottle was placed in a constant temperature water bath at (37.3 ± 0.20) °C for 10 min to equilibrate the polymer solution to completely form a gel. Carefully add 37.5 °C, 1 mL of PBS as the release medium, and shake at 0× *g* vs. 0.035× *g* in a constant temperature shaker at 37.5 °C, respectively. Pour out all the release medium at the set time, respectively, blot the inner and outer surfaces of the vessel with filter paper, weigh and record quickly, and then re-equilibrate in the constant temperature water bath shaker for 10 min, and then replenish the release medium by 1.0 mL. Do so repeatedly until the gel is completely dissolved. Three cillin bottles were set up for each test and the average value was taken. The difference in sample mass at adjacent time points was the amount of gel dissolution during this period. The experimental procedure was repeated three times, and the mass difference existing between adjacent time points was the hourly amount of dissolution of the gel, from which the cumulative dissolution rate X was calculated as:

X = (W_n_ − W_0_)/W × 100%

In this equation, W_0_ is the initial total mass (g) of this gel and the cillin bottle, W_n_ is the total mass (g) of the gel and the cillin bottle for the n time, and W is the gel mass (g). The cumulative lysis rate (X) of the in situ gel of the “Jifang E” was plotted against time (t) to obtain the lysis curve of this gel. The results were repeated three times and the results were averaged.

The drug-release medium obtained at different time points was volume fixed, and the drug concentration was measured by a spectrophotometer and the cumulative drug release rate (Q) was calculated, and the drug–release curve was obtained by plotting Q and t. The kinetics of film-free dissolution were regressed against the cumulative drug release rate.

M and Q were calculated as follows: M = Et/E × 100%, where Et is the cumulative gel dissolution at time t and E is the initial gel mass; Q = Rt/R × 100%, where Rt is the cumulative drug release at time t and R is the theoretical drug loading.

With membrane release, 5 mL of “Jifang E” in situ gel was removed and placed in a dialysis bag, and the ends were tied with cotton thread. A 250 mL conical flask was filled with 200 mL of pH 7.36 PBS and antioxidant as the release medium. The dialysis bag with “Jifang E” in situ gel was incubated at 37.5 °C for 10 min to allow complete gelation of “Jifang E” in situ gel. They were then placed in a conical flask, heated in a constant temperature shaker at 37.5 °C, and shaken at 0.035× *g*. At specific times, 3 mL of release medium was taken by syringe and the same volume of fresh release medium (37.5 °C) was added. The volume of release medium obtained at different points was fixed and measured by UV spectrophotometer to calculate the release rate of “Jifang E”. Each group of experiments was repeated three times.

### 2.8. Acute Toxicity Test in Mice for “Jifang E” Sustained-Release Injection

Seventy SPF mice were randomly divided into 7 groups of 5 males and 5 females. Five control groups received hypodermic injections of “Jifang E” at doses of 325 mg/kg, 500 mg/kg, 770 mg/kg, 1183.4 mg/kg, and 1820.6 mg/kg; the remaining two groups were blank and saline groups, respectively. The doses of both groups were the same as the highest dose of the experimental group. The mice were observed continuously for 7 days, and the time of death, number, and status were recorded. During the period, the dead mice were dissected and the organs were observed visually for lesions, and the lesioned organs were observed histologically. After 7 days, all mice were dissected to observe the organs, and the LD_50_ of the “Jifang E” sustained-release injection was calculated by the modified Kou’s method.

LD_50_ and 95% confidence limits of LD_50_ were calculated:

LD_50_ = log^−1^[X_m_ − i(∑P − 0.5)]

SD = i·(∑P − ∑P^2^/n − 1)

95% confidence limits of LD_50_ = log^−1^(logLD_50_ ± 1.96 × SD)

where X_m_ is the logarithmic value of the maximum dose; P is the mortality rate of animals in each group; ∑P is the sum of mortality rates of animals in each group; and i is the group spacing.

### 2.9. Biocompatibility and Muscle Irritation Test of “Jifang E” Sustained-Release Injection

Three New Zealand rabbits, weighing 2.5 ± 0.7 kg, were randomly selected for subcutaneous injection of the “Jifang E” sustained-release injection into the back of two rabbits and 0.9% NaCl into one rabbit; for the muscle stimulation test, the self-control method was chosen, i.e., all rabbits were injected with 0.9% NaCl into the quadriceps muscle of the left hind limb and the “Jifang E” sustained-release injection into the quadriceps muscle of the right hind limb. After 48 h, all rabbits were euthanized, and the quadriceps muscles of both legs and the subcutaneous tissue of the back were cut longitudinally to observe any changes, the rabbits were graded according to the criteria of the stimulus response level in Table 1, and the muscle stimulus response level less than or equal to 2 could be provided for intramuscular injection. Muscle and subcutaneous tissues at the injection site were selected, soaked in paraformaldehyde, and sections were made for histopathological observation.

### 2.10. Pharmacokinetic Test of “Jifang E” Sustained-Release Injection in Mice

Forty SPF Kunming white mice, weighing 25–30 g, were subcutaneously injected on the back with the “Jifang E” sustained-release injection according to 150 mg/kg·bw. Before and 0.5 h, 1 h, 2 h, 4 h, 8 h, 12 h, 24 h, 48 h, 72 h, 96 h, 120 h, 144 h, 168 h after administration, blood samples were collected from the orbital canthal plexus of each mouse using glass capillaries containing heparin at 0.4 mL, placed in a blood collection tube containing heparin, centrifuged at 1000× *g* for 10 min, aspirated the upper layer of plasma, placed in centrifuge tubes, and stored at −20 °C. The blood concentration of “Jifang E” was spiked according to the instructions of the kit, then the wavelength was set according to the requirements and detected in the enzyme marker, and the concentration of “Jifang E” in plasma was calculated by substituting the formula as in the instructions of the kit.

### 2.11. Statistical Analysis

The calculated concentration data of “Jifang E” were entered into DAS 2.0 (Chinese Pharmacological Association, Hefei, Anhui Province, China) software, and the optimal atrial model was obtained by intelligent analysis, the AIC was the smallest and R^2^ was the closest to 1. GraphPad Prism 8.0 was used to plot blood concentration–time curves, and all results were expressed as mean ± standard deviation.

## 3. Results

### 3.1. Formulation Development for “Jifang E” Sustained-Release Injection

**Figure 1:**
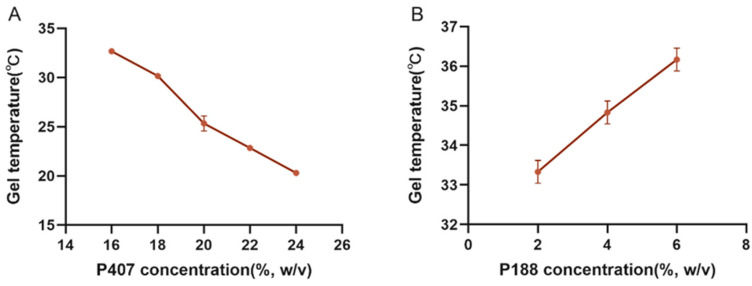
The effect of P407 and P188 on the gelling temperature. (**A**) is the effect of P407 on the gelling temperature; (**B**) is the effect of P188 on the gelling temperature when the concentration of P407 is fixed at 20%.

Poloxamer 407 was chosen as a sustained-release excipient because it is a substance with amphiphilic properties of polyethylene oxide (PEO) and polypropylene oxide (PPO), which can be gelled below physiological temperature at concentrations of 15% to 30% (*w*/*v*), and the gelling temperature gradually decreases as the concentration increases (Figure 1A). The “Jifang E” is encapsulated in the gel and is released through the pore structure in the gel with the solubilization of the gel. The relative molecular mass of hydrophilic polyoxyethylene (PEO) accounted for a higher percentage of the gelling temperature with the increase in concentration of Poloxamer 188 compared to Poloxamer 407 (Figure 1B); polyethylene glycol 4000 was also added as a binder in the formulation to optimize the solution formulation; polyethylene glycol is water soluble and the hydrophilic structure in it also elevates the gelling temperature (Table 2); and vitamin C was added to the solution as an antioxidant to prevent oxidation of “Jifang E”. Vitamin C is also hydrophilic, which also raises the gelling temperature (Table 3), and it was concluded that “Jifang E” would reduce the viscosity of the gel solution and increase the gelling temperature (Table 4). Through the screening of different formulations, the optimal ratio of P188 (6%), Vitamin C (1%), PEG4000 (0.5%), and “Jifang E” (10%) was developed. 

### 3.2. Detection of Micelle Hydrodynamic Diameter and Its Distribution

The interaction of the drug, as well as additives, with micelles was studied by dynamic light scattering (DLS), and the effects of temperature, micelle composition, and the addition of drug and additives on the hydrodynamic diameter and particle size distribution parameters of the micelles were observed. The results are summarized in Table 5.

The results showed that the group formulations used typically showed three peaks at either 25 °C or 37 °C, but the particle size distributions differed. For example, the highest percentage of particle size distribution was 618.97 nm at 25 °C for the micellar solution containing only P407, while the highest percentage of particle size distribution was 19.49 nm at 37 °C. As hydrodynamic diameter of the micelles decreased, and there was a peak of 3394.33 nm at 37 °C, the percentage was lower, which indicated that there were some large particle impurities in the micellar solution. Moreover, the PDI values at both temperatures are above the applicable analytical range for dynamic light scattering with a very wide size distribution. As for the binary system P407/P188, although it also has three peaks, it has a particle size distribution with a larger percentage difference, and this difference is more significant at 37 °C, and the PDI values are more in line with the dynamic light scattering analysis. In addition, the micelle size of all groups decreased with the increase of temperature, and the whole micelle system was more stable with the addition of P188, additives, and drugs, and the particles were uniformly dispersed.

### 3.3. Rheological Analysis

**Figure 2:**
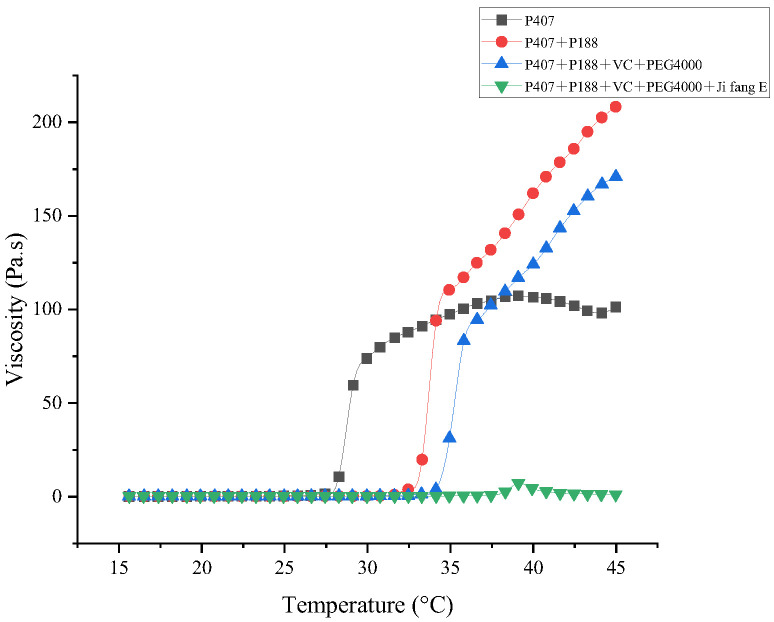
Viscosity changes of different formulations with increasing temperature.

The effects of the rheological properties of different prescriptions on drug delivery, drug release, and in situ adaptability of the deposited drug were evaluated by viscosity and viscoelasticity, respectively. Our results showed (Figure 2) that the viscosity of different composites of the formulation increased with the increase of temperature, which is due to the fact that the increase of temperature induced the change of each composite from the solution state to the gel state. This indicates that P188 can not only increase the gelation temperature of P407, but also increase the gel strength of P407; after adding additives, the gelation temperature increased and the viscosity decreased, which was caused by the hydrophilic structure of the additives; the addition of “Jifang E” further altered the viscosity. This is due to the fact that the addition of “Jifang E” affects the structure of the gel matrix. In addition to lowering the viscosity and increasing the gelation temperature, the addition of “Jifang E” also caused salt precipitation in the gel matrix and changed the overall structure of the gel as determined by the nature of the drug itself. 

**Figure 3:**
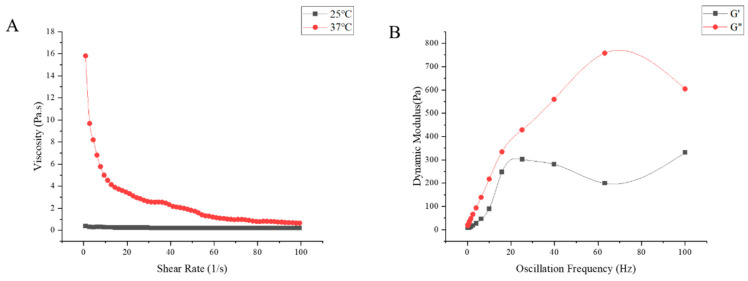
The viscosity changes with shear rate at 25 °C and 37 °C (**A**) and the dynamic modulus changes with oscillation frequency at 37 °C (**B**) of “Jifang E” sustained-release injection were observed.

In addition, the sustained-release in situ gel “Jifang E” exhibits shear thinning properties, which facilitates injection at high shear rates. The viscosity of the “Jifang E” sustained-release in situ gel decreased with increasing shear rate, indicating a structured pseudoplastic fluid (Figure 3A). At the temperature of 25 °C, the viscosity of “Jifang E” sustained-release in situ gel did not change much with the increase of shear rate, because at 25 °C, “Jifang E” sustained-release in situ gel was always in the liquid state; at the temperature of 37 °C, the sustained-release in situ gel had formed a gel, and the viscosity of the in situ gel was not changed. In situ gel has formed a gel, viscosity increases, with the increase of shear rate viscosity decreases, which indicates that with the increase of shear rate, “Jifang E” sustained-release in situ gel gradually becomes thin, the decrease in viscosity at high shear rates indicates that “Jifang E” sustained-release in situ gel has good thixotropic properties, suggesting that it may not cause discomfort in the body because its shape can change to accommodate deformation of surrounding tissues during body movement. 

**Figure 4:**
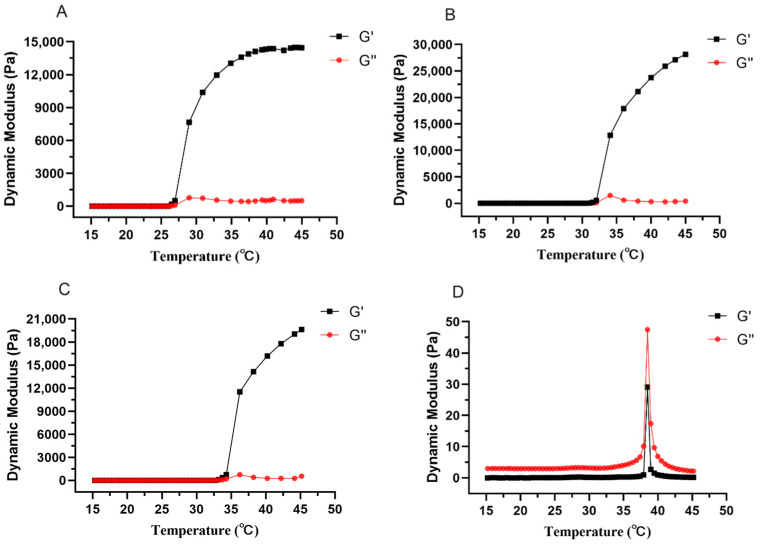
The changes of elastic modulus and viscous modulus of different formulations with the increase of temperature. The prescription for (**A**–**D**) is P407, P407 + P188, P407 + P188 + additive, P407 + P188 + additive + “Jifang E”.

Viscoelasticity consists of viscous modulus (G″) and elastic modulus (G′), which represent the properties of liquid and solid, respectively. Figure 3 represents the changes of viscous modulus and elastic modulus of different prescriptions with increasing temperature, before the addition of drug, the elastic modulus of all prescriptions were increasing and significantly higher than the viscous modulus after being equal to the viscous modulus (Figure 4A–C), the temperature at which they are equal is the gelling temperature of the prescriptions, and the elastic modulus is greater than the viscous modulus indicating that the prescriptions are very elastic and can form gel at physiological temperatures. After adding the drug, the elastic modulus was always smaller than the viscous modulus with the increase of temperature (Figure 4D), and Figure 3B shows the variation of G′ and G″ with the oscillation frequency of “Jifang E” sustained-release injection gel (37 ± 0.5 °C). The results show that the viscous modulus is higher than the elastic modulus in the frequency range of 10^−1^ to 10^2^ Hz, indicating that the formed gel is more viscous than elastic. When the gelling temperature was tested, the prescriptions to which the drug was added were able to form gels at physiological temperatures, probably because the drug disrupted the structure of the gel matrix, resulting in a dramatic change in its rheological properties, so that the rheological parameters would not match those observed by the eye.

### 3.4. Morphological Analysis by Electron Microscopy

**Figure 5:**
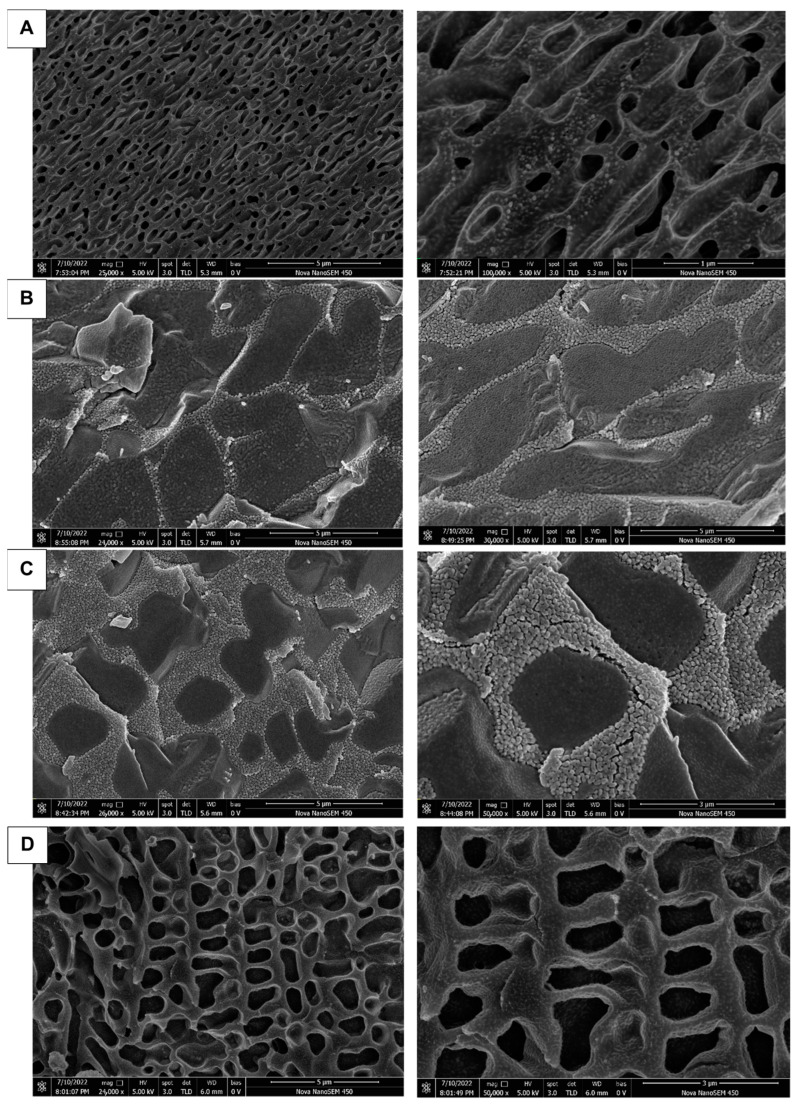
Characterization under SEM after gelation of different formulations. The prescription for (**A**–**D**) is P407, P407 + P188, P407 + P188 + additive, P407 + P188 + additive + “Jifang E”.

From Figure 5A–D it can be seen that the pore structures were all after gelling. With the addition of P188 and additives, the stability of the gel decreases and the bioadhesion decreases, probably because of the high content of the hydrophilic structure of P188 and additives, which leads to the unstable structure after gelling (Figure 5B,C); while the higher concentration of P407 has a lower gelling temperature and higher lipophilic structure with a higher molecular content, it is more stable and forms a dense net-like structure after gelling (Figure 5A); “Jifang E” contains metal ions, although it will destroy the structure of the gel, but the impact of emulsification on the gel will form a milky gel mass, increasing the gel viscosity and resulting in increased stability (Figure 5D). Milky gel mass will generally be at the bottom of the liquid, thus before its use the container should be turned upside down and shaken evenly. After “Jifang E” is injected into the body and gelled, the drug will be released through the pores, and the granular material distributed on the surface of the gel structure is hydrophilic.

All life sciences and many materials’ science samples contain liquid components. The natural drying method in air is unacceptable due to its severe wrinkling and distortion. Conventional treatments (such as critical point drying or freeze-drying methods) still have some degree of wrinkling and distortion, loss of soluble components, slow preparation processes, and other disadvantages, and cannot be used for liquid and semi-liquid samples and samples sensitive to electron beam. Therefore, in using the Cryo-SEM method, the above-mentioned disadvantages can be completely overcome; the sample does not need to go through drying processes such as critical point drying, direct freezing observation occurs without damaging the sample structure, it is easy to operate, and the observed image is clearer.

### 3.5. In Vitro Dissolution and Drug Release Assays

**Figure 6:**
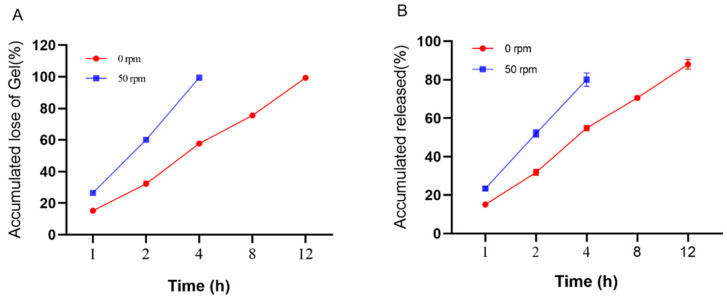
In vitro no membrane dissolution (**A**) and drug release (**B**) of “Jifang E” sustained-release injection at 0, 50 rpm as a function of time.

**Figure 7:**
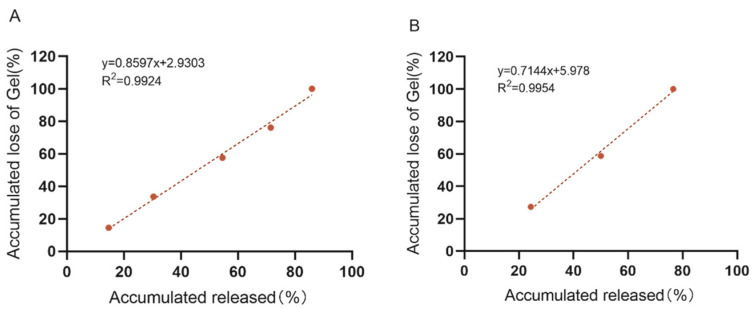
In vitro no membrane dissolution and drug release standard curves of “Jifang E” sustained-release injection at 0 rpm (**A**) and 50 rpm (**B**).

The release pathways of temperature-sensitive gels using Poloxamer 407 as a sustained-release coagent include gel dissolution and gel pore release. In order to maintain a stable and effective drug concentration and not to cause side effects to the body due to high drug concentration, an in vitro release and dissolution study was conducted to estimate the in vivo release characteristics of the “Jifang E” sustained-release injection. The main simulated drug release pathway in the filmless in vitro drug release is gel dissolution. At 0 rpm, the drug-containing gel was completely dissolved at 12 h and the corresponding drug release time was at 12 h, while at 50 rpm, the drug-containing gel was completely dissolved at 4 h and the drug release time was at 4 h (Figure 6). This indicates that the oscillation frequency has an effect on the slow release of the drug, and the higher the oscillation frequency, the faster the drug release rate. In filmless drug release, the drug-sustained release gel is in direct contact with the release medium, the slow-release gel structure is washed out by the aqueous phase, the structure is loose, the drug is originally small in molecular weight, and it is water soluble, so the drug release rate is accelerated. Moreover, the drug is easily oxidized in the liquid, and the release rate will be deviated. The correlation analysis between dissolution rate and drug release rate was performed (Figure 7A,B), and the correlation between dissolution rate and drug release rate was R^2^ = 0.9924 and R^2^ = 0.9954 at 0 rpm and 50 rpm, respectively, indicating that there is a correlation between the dissolution rate and drug release rate of drug-containing sustained-release gel, and the drug is released with gel dissolution in filmless in vitro drug release. 

**Figure 8:**
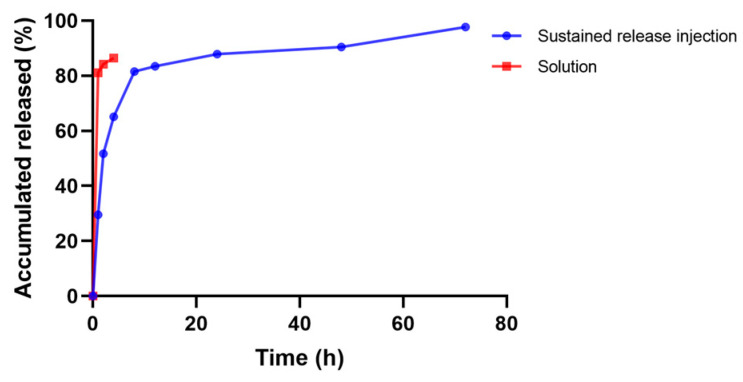
Drug release profiles of “Jifang E” sustained-release injection.

In vitro drug release with a membrane mainly simulates the release of drug through the pores of the gel. The drug-containing gel is not in direct contact with the drug-release medium, but is separated from the drug-release medium by the dialysis bag, which has a retention molecular weight (MD 8000–12,000), and the molecular weight of the drug is small and will be easily released into the drug-release medium through the dialysis bag. The sustained release gel of “Jifang E” can be released continuously for 3 days and has a sudden release effect within 12 h (Figure 8), which is due to the fact that “Jifang E” is a water-soluble drug with a small molecular weight, which can be released faster by itself, and the gel structure becomes loose after coming into contact with a large amount of drug-release medium. The dense structure of the gel was destroyed, which accelerated the release of the drug. After 12 h, the drug was released slowly, which is consistent with the release pattern of water-soluble drugs in the sustained-release gel. Compared with the “Jifang E” sustained-release injection, the aqueous solution of “Jifang E” had a release rate of more than 80% at 1 h (Figure 8), and both injectable dosage forms did not release completely, which may be due to the fact that the drug itself is easily oxidized. In general, “Jifang E” sustained-release injection has a certain slow-release effect.

### 3.6. Acute Toxicity Test in Mice for “Jifang E” Sustained-Release Injection

After the “Jifang E” sustained-release injection, all groups of mice showed depressed activity; they had poor appetites, their movement was slow, and they were insensitive to external stimuli. The mice in the high-dose group were the first to show clinical signs, which included restlessness, unsteady gait, some mice jumping up, heavy breathing, and general convulsions. The mice in the low-dose group were sedentary and did not feed or drink after the “Jifang E” sustained-release injection. Mice in the 325 mg/kg group recovered their mental state and appetite after 4–5 h. Two mice in the 500 mg/kg group died after 24 h. Mice in the 770 mg/kg group started to die from 6 h and 5 mice died after 24 h. The number of male mice died was higher. Mice in the 1820.6 mg/kg group started to die from 2 h and all the mice in the group died after 24 h. The number of dead mice in the 1820.6 mg/kg group was nearly the same for both sexes. The mice that did not die after 24 h of injection recovered their spirit and appetite and healed well. Gross autopsy was performed on the dead mice and surviving mice, and there were no obvious lesions in the organs by visual observation. The mice in the blank lysate and saline groups did not have any of the above abnormal signs, and their feeding, drinking, mental appetite, and locomotor appearance were normal, and there were no dead mice. There were no obvious lesions in the organs under visual observation.

The mortality of mice in each group was counted after poisoning as shown in Table 6, the LD_50_ = 828.323 mg/kg, SD = 0.0448, and the 95% confidence limit of LD_50_ was 828.323 mg/kg (676.706–1013.911 mg/kg) for the “Jifang E” sustained-release injection using the modified Kou’s method.

### 3.7. Biocompatibility and Muscle Irritation Test of “Jifang E” Sustained-Release Injection

It was observed that all three rabbits were active, fed and watered normally, alert, and had no adverse reactions after administration. There were no obvious ocular changes in the quadriceps muscle on both sides of the rabbits injected with saline and the “Jifang E” sustained-release injection. When the rabbits were euthanized 48 h after intramuscular injection, both quadriceps muscles were removed, and a slight swelling and congestion were observed visually on the side of the quadriceps muscle injected with the “Jifang E” sustained-release injection, and no local hard mass or necrotic tissue was found. The stimulation response level was between 1 and 2, indicating that the “Jifang E” sustained-release injection could be used for intramuscular injection. 

**Figure 9:**
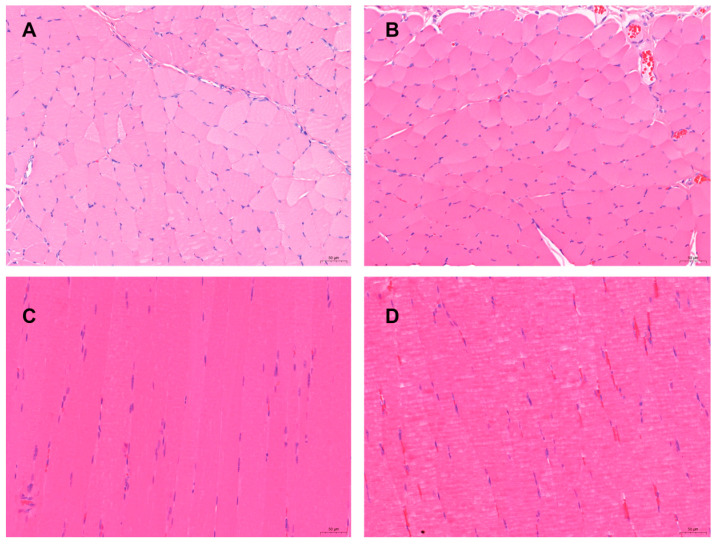
The pathological changes of “Jifang E” sustained release injection were observed by intramuscular and subcutaneous injection. (**A**): the subcutaneously muscle tissue of the back without administration; (**B**): the subcutaneously muscle tissue of the back with administration, no obvious pathological changes were observed; (**C**): the quadriceps muscle tissue without administration (the left quadriceps muscle tissue); (**D**): the quadriceps muscle tissue with administration (the right quadriceps muscle tissue), no obvious pathological changes were observed. HE × 200.

The quadriceps muscle and the subcutaneous muscle tissues at back at the administration site showed no obvious changes with the naked eye and only slight congestion. Pathological histological observation showed that the quadriceps muscle at the administration site was cut longitudinally with the subcutaneous tissue, compared with the muscle tissue without administration (Figure 9A,C) no obvious pathological changes were observed: the muscle fiber morphology was normal, although occasionally some muscle fibers had uneven cytoplasmic staining, without wavy, vacuolar, or fatty degeneration, no obvious necrosis was observed, and no obvious infiltration or proliferation of inflammatory cells were observed in the stroma (Figure 9B,D).

### 3.8. Pharmacokinetic Test of “Jifang E” Sustained-Release Injection in Mice

**Figure 10:**
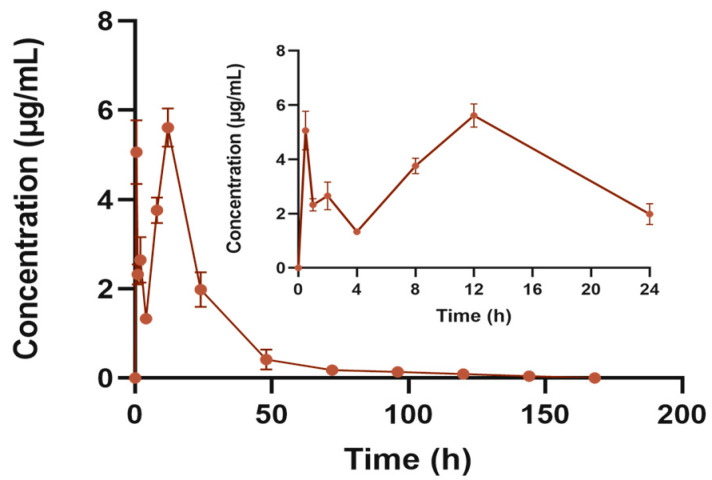
Blood concentration–time curve of “Jifang E” sustained release injection. The subgraph shows the plasma concentration–time curve at 24 h.

After a single subcutaneous “Jifang E” sustained-release injection, blood was collected according to the time point and the concentration of the drug in plasma was measured. As shown in Figure 10, the blood concentration of the “Jifang E” sustained-release injection showed a double-peak trend over time, probably because the sustained-release injection gradually became jelly-like when it came into contact with the subcutaneous tissue under the effect of body temperature, and in this process the drug was easily released in the flowing sol, which was the reason for first peak. And then, the drug is slowly released by the sustained-release matrix encapsulated in the gel, and with the movement of the animal, as well as the friction and shearing between the muscle and skin at the injection site, the viscosity of the jelly-like sustained-release injection decreases and there is more release of the drug again, which is the reason for the second peak. 

The results of the pharmacokinetic parameters are shown in Table 7, and a single subcutaneous injection of “Jifang E” at a dose of 150 mg/kg was administered. The concentration of “Jifang E” in plasma reached a maximum of 5.609 ± 0.43 μg/mL at 12 h after a single subcutaneous “Jifang E” sustained-release injection, and then decreased, with the concentration of the drug decreasing rapidly on the first day to 0.4 μg/mL at 48 h. In order to ensure the effect of the drug, repeated dosing is required, but repeated dosing can cause accumulation of toxicity in the body, so the dosing repeated administration requires repeated discretion. The AUC_(0−∞)_ of the area under the curve was 131.002 ± 14.849 μg/mL·h, the relative bioavailability against “Jifang E” solution is 63.17%. The average retention time of the “Jifang E” sustained-release injection in vivo was approximately 24 h with a half-life of 32.821 ± 16.798 h.

## 4. Discussion

Previous studies have shown that the gelation temperature decreases as the proportion of P407 increases, the gelation temperature increases as the concentration of P188 increases, and the gelation temperature increase also correlated with the increasing concentration of polyethylene glycol [16]. The concentration–gelation temperature trend of P407 in this study is consistent with the results of previous studies [17]. However, the addition of P188 and P407, together with the addition of “Jifang E”, decreased the gelation temperature, probably because the metal ions in “Jifang E” form emulsion gels by ionization, which interact with hydrophobic groups, thus lowering the gelling temperature. Moreover, the dissolution of “Jifang E” in liquid increases the rate of oxidation. Based on this observation, the content of “Jifang E” should not exceed 10% (*w*/*v*). The gelling temperature of temperature-sensitive gels is not only related to the nature of each formula, but also closely related to the area of the gel contact surface and the friction of the contact surface. The insoluble Ber increases the entanglement strength of 407, which decreases the rate of sol–gel kinetics, thus decreasing the gel temperature, enhancing the adhesion of the cornea, which in turn decreases the rate of gel erosion and drug release. The addition of hydroxy propyl methyl cellulose (HPMC) helped to enhance the gel strength [18]. Poloxamer 188 increased the gel temperature and accelerated drug release, and the additive NaCl increased the gel temperature and gel strength [19].

The micelle size of the temperature-sensitive gel system based on poloxamer decreases with increasing temperature, and this trend was also shown in this study. It has been shown that there is a relationship between this parameter and the dehydration of PPO units as well as the viscosity of the formulation, which favors the formation of colloidal systems with homogeneous spherical micelles [20]. Injectables with Poloxamer 407 as a sustained release matrix showed Newtonian fluid characteristics with increasing shear rate at <30 °C and pseudoplastic fluid characteristics after >30 °C [21], which is consistent with the results obtained in this study. When the external temperature is lower than the sol–gel transition temperature, G′ is less than G″ and the solution is in the sol state. As the temperature increases, the hydrogel G′ increases rapidly and is higher than G″, indicating that the hydrogel has completed the sol–gel transition and exhibits higher elasticity [22]. In this study, the formulation before the addition of the drug was consistent with this phenomenon, but the addition of “Jifang E” changed this characteristic, and the results showed that with the addition of the drug, the elasticity of the gel decreased and showed more viscosity, while at approximately 37.5 °C both had a significant increase, and then both decreased after the temperature increased, which indicated that, at this temperature, both the elasticity and viscosity of the gel increased, but the subsequent decrease may be due to the destruction of the structure of the gel by the drug. As the temperature increased, the viscosity of each formulation increased, with low viscosity before gelling, which facilitated injection and increased viscosity at higher temperatures, which facilitated a slow release of the drug [23]. Hydrogel networks contain porous structures in certain sizes known as the mesh sizes. Controlling the mesh size in the hydrogel network is essential for sustained therapeutic drug delivery [24]. Ningxia Xu [25] investigated the morphology of different concentrations of Poloxamer 407 with 188 under scanning electron microscopy, and showed that different concentrations of Poloxamer 407 with 188 formed a mesh-like pore structure after gelling, the pore structure was not uniform, and these pores could be used as a channel for the diffusion of drugs, bioactive molecules, and other small molecules.

Guo Junguo [26] used both as the extended-release matrix, and the in vitro release showed that the extended-release injection could be released for 5 h. The reason for the large difference in in vitro release with the same sustained-release injection is not clear. It is possible that the in vitro release method used is different and the nature of the drug is different. In situ thermally reversible injectable gel with a controlled release of simvastatin (SMV) was made from poloxamer (PM) and methylcellulose (MC). PM 25% and MC 5% at 37 °C formed an ideal temperature sensitive injectable gel for SMV with an in vitro release time of 10 days [27].

A single intraperitoneal injection of 1 g/kg of Poloxamer 407 has been shown to cause hyperlipidemia in rats [28]. It has been shown that the maximum oral tolerance (MTD) of substrate given by gavage in mice is >16 g/kg and the LD_50_ is >6.0 g/kg; the LD_50_ of intraperitoneal injection in mice is 3.66 g/kg (P407 solution), 7.74 g/kg (P188 solution), and 5.62 g/kg (P407/P188 solution); the MTD of substrate given vaginally in rats is >2.4 g/kg [29]. In this study, the concentrations of Poloxamer 407 and Poloxamer 188 were 0.44 g/kg and 0.11 g/kg, respectively, and the mixed concentration of both was 0.55 g/kg, and the release was slow by subcutaneous injection, which was somewhat less toxic compared with intraperitoneal injection. Therefore, concentrations of Poloxamer 407 and 188 used in this study were within the safe range. In the acute toxicity test of “Jifang E” mice by oral gavage, the mice first showed depression, they had a loss of appetite, closed eyes, were lying still, were curled up, and had an arched back, and this behavior was followed by restlessness and unsteady gait, followed by paroxysmal whole-body trembling, then difficulty in breathing, and death. This symptomology exhibition differs from the acute toxicity of “Jifang E”. This is the same as the clinical signs of mice in the acute toxicity test of the “Jifang E” sustained-release injection. The LD_50_ of “Jifang E” was 2808 mg/kg, which is a low-toxicity substance according to the acute toxicity dose classification of chemical substances [30]. However, there are other substances in the drug formula, and the sustained-release excipients account for a large proportion, which makes “Jifang E” be released slowly, the toxic effect on the test animals will be reduced, and the time of adverse reactions will be prolonged. Moreover, after subcutaneous “Jifang E” sustained-release injection, mice initially showed depression and loss of appetite, but gradually recovered after 24 h. This indicates that a one-time administration of the appropriate concentration of the “Jifang E” sustained-release injection will not cause long-term toxic reactions in the body.

After subcutaneous injections, the drug is slowly released by the action of body temperature by forming a gel reservoir at the injection site, which is biocompatible, non-irritating, and has a pH close to neutral, and the other substances in the prescription, such as Poloxamer 188 and Poloxamer 407, have similar properties, and the only difference is the size of the relative molecular weight and the proportion of each of PPO and PEO, so the safety is better, as confirmed by previous studies [31,32]. Polyethylene glycol and vitamin C account for a small percentage and both are also non-irritating. The slight swelling and congestion of the muscle and subcutaneous tissue at the site of administration are mainly due to the irritation of “Jifang E”. Therefore, the amount of the drug is kept as small as possible to reduce the irritation of the subcutaneous tissue.

The drug with the use of Poloxamer 407 in its formulation as a sustained-release matrix can be released in vivo for 4–7 days, which is consistent with the veterinary clinical dosing cycle (3–5 days) [33]. The temperature-sensitive gel for the injection of borneolimycin hydrochloride prepared by compounding with Poloxamer 407 as the main matrix material can be released continuously for more than 48 h in rats [34]. A co-blended gel system consisting of a warm-sensitive hydrogel with an optimal ratio of Poloxamer 407 and 188 mixed as a matrix for the treatment of arthritis can be released continuously for 28 days [10]. The results of this study showed that the sustained-release injection of “Jifang E” could be released for approximately 5 days, which is consistent with the sustained-release injection of other drugs using Poloxamer 407 as the in situ gel matrix mentioned above, and is similar to the results of the in vitro membrane release test of 3 days. The absorption half-life T_1/2_ was 32.821 ± 16.798 h. The peak blood concentration was reached at 12 h with a C_max_ of 5.609 ± 0.43 μg/mL and an area under the drug–time curve AUC_(0−∞)_ of 131.002 ± 14.849 μg/mL·h. The results of the test for “Jifang E” solution (at a dose of 20 mg/kg) showed that the half-life T_1/2_ of “Jifang E” was (3.15 ± 0.180 h), and the peak blood concentration was (5.65 ± 0.77) h. The C_max_ was (4.98 ± 0.68) μg/mL, and the AUC was (27.65 ± 3.54) μg/mL·h [35]. Compared with the bulk drug, the half-life, peak time, peak concentration, and area under the drug–time curve of the “Jifang E” sustained-release injection have great differences. The gastrointestinal absorption will go through the first-pass metabolism and the hepatic and intestinal circulation will consume part of the drug, although the drug release is slower, the drug is directly absorbed into the blood through the capillaries of the subcutaneous tissue, compared with the oral route of drug delivery, subcutaneous injection absorption will be faster and the peak concentration will be higher. Therefore, our results suggest that “Jifang E” solution when injected subcutaneously, can be absorbed faster than oral administration. At present, “Jifang E” is mainly used for the prevention of livestock poisoning in pill form taken orally, and the drug is absorbed through the stomach and intestines. The dose of 20 mg/kg·bw is fed once a day with water in bulk, and two pills are fed to each sheep, each containing 10 g of “Jifang E”, which can protect sheep for 60–70 days [36]. 

## 5. Conclusions

In this study, we have shown that the temperature-sensitive gel based on poloxamer can achieve injectability and sustained release of “Jifang E” in vivo. It is safe, has good biocompatibility, and can maintain an effective blood drug concentration for 5 days. It forms a gel at physiological temperature at the injection site and does not form non-degradable material. Based on these results, it can be concluded that the sustained-release injection of “Jifang E” based on the poloxamer, providing a new method for preventing swainsonine-containing plants poisoning grassland livestock, has great potential for wide application.

## Figures and Tables

**Table 1:** Criteria for determining the magnitude of stimulus response.

Reaction Order	Stimulus Response
1	No significant reaction at the site of drug
2	Mild congestion at the site of drug, less than 0.5 cm in diameter
3	Moderate congestion at the site of drug, less than 1 cm in diameter
4	Highly congested, red and swollen drug site with muscle degeneration
5	Appearance of brown muscle degeneration and necrosis with a diameter of 0.5 cm or less
6	Severe muscle degeneration with extensive necrosis

**Table 2:** Effect of Vitamin C on gelling temperature.

VC Concentration (%)	Gel Temperature (°C)
0	30.07 ± 0.33
1	34.83 ± 0.62
2	37.40 ± 0.65

Other formulas are P407 (24%) + P188 (6%) + ”Jifang E” (10%).

**Table 3:** Effect of PEG4000 on gelling temperature.

PEG4000 Concentration (%)	Gel Temperature (°C)
0	30.07 ± 0.33
0.5	32.50 ± 0.41
1	38.17 ± 0.62

Other formulas are P407 (24%) + P188 (6%) + Jifang E (10%) + VC (1%).

**Table 4:** Summary of effects of P407, P188, and drugs on gelling temperature.

Prescription	Gel Temperature (°C)
20%P407 + 2%P188 + 10%“Jifang E”	>37
20%P407 + 4%P188 + 10%“Jifang E”	>37
20%P407 + 6%P188 + 10%“Jifang E”	>37
22%P407 + 2%P188 + 10%“Jifang E”	>37
22%P407 + 4%P188 + 10%“Jifang E”	>37
22%P407 + 6%P188 + 10%“Jifang E”	>37
24%P407 + 2%P188 + 10%“Jifang E”	>37
24%P407 + 4%P188 + 10%“Jifang E”	>37
24%P407 + 6%P188 + 10%“Jifang E”	33.07 ± 0.33

**Table 5:** Particle size and average distribution of different formulations at room temperature (25 °C) and body temperature (37 °C).

Formulations	25 °C		37 °C	
	Hydrodynamic Diameter (nm)	Average Distribution (%)	Hydrodynamic Diameter (nm)	Average Distribution (%)
P407	618.97 ± 32.41	73.97	19.49 ± 0.77	54.73
	40.30 ± 1.67	19.50	576.13 ± 86.05	43.80
	5.17 ± 0.47	5.57	3394.33 ± 2400.73	1.47
P407 + P188	60.01 ± 1.58	78.10	27.90 ± 0.69	95.45
	5.55 ± 0.10	20.07	5.11 ± 0.71	4.6
	4658.00 ± 397.05	1.87	-	-
P407 + P188 + VC + PEG4000	51.41 ± 0.97	66.90	30.99 ± 1.19	88.83
	212.27 ± 293.11	15.20	378.54 ± 230.22	6.73
	463.69 ± 332.39	14.43	1553.57 ± 2193.04	3.13
P407 + P188 + VC + PEG4000 + “Jifang E”	47.06 ± 2.01	73.7	27.06 ± 0.92	93.00
	1409.03 ± 504.55	15.03	4471.67 ± 506.89	5.7
	5.58 ± 10.53	10.53	2.27 ± 3.20	1.3

**Table 6:** Results of acute toxicity test on mice from “Jifang E” sustained-release injection.

Group	Dose	Logarithmic	Number of Animal	Number of Death	Mortality Rate	Survival Rate	p·q
	(mg/kg)				(p)	(q)	
1	325	2.5118	10	0	0.0	1.0	0.00
2	500	2.6989	10	2	0.2	0.8	0.16
3	770	2.8864	10	5	0.5	0.5	0.25
4	1183.4	3.0731	10	7	0.7	0.3	0.21
5	1820.6	3.2602	10	10	1.0	0.0	0.00
	i = 0.08					Σp = 2.4	

**Table 7:** Pharmacokinetic parameters of “Jifang E” sustained-release injection.

Pharmacokinetic Parameters	Parameter Values
t_1/2z_ (h)	32.821 ± 16.798
AUC_(0−t)_ (μg/mL·h)	128.274 ± 15.091
AUC_(0−∞)_ (μg/mL·h)	131.002 ± 14.849
MRT_(0−t)_ (h)	23.246 ± 1.22
MRT_(0−∞)_ (h)	26.231 ± 2.527
T_max_ (h)	12
C_max_ (μg/mL)	5.609 ± 0.43

## Data Availability

Not applicable.
